# Deep Phenotyping of F64L Mutation in a Multicentric Cohort of Patisiran‐Treated Hereditary Transthyretin Amyloidosis Patients (Patisiranitaly)

**DOI:** 10.1111/ene.70657

**Published:** 2026-06-01

**Authors:** Marco Ceccanti, Pietro Guaraldi, Angela Romano, Giovanni Antonini, Alessandro Barilaro, Chiara Briani, Marco Burattini, Micol Gianoli, Giulia Carlini, Vittoria Cianci, Marco Currò Dossi, Daniela Di Lisi, Antonio Di Muzio, Adele Ratti, Massimiliano Filosto, Sabrina Gasverde, Chiara Gemelli, Luca Gentile, Mariangela Goglia, Luca Leonardi, Simone Longhi, Antonio Lotti, Fiore Manganelli, Anna Mazzeo, Sofia Maria Augello, Giammarco Milella, Giuseppina Novo, Davide Pareyson, Silvia Fenu, Giovanni Palumbo, Cristina Petrelli, Loris Poli, Luca Guglielmo Pradotto, Massimo Russo, Alessandro Salvalaggio, Maria Ausilia Sciarrone, Luigi Sellitti, Matteo Tagliapietra, Stefano Tozza, Mariagiovanna Castiglia, Mara Turri, Lorenzo Verriello, Cristina Chimenti, Francesca Vitali, Filippo Brighina, Nicasio Rini, Maurizio Inghilleri, Roberto D'Angelo, Domenico Abelardo, Chiara Cambieri, Laura Libonati, Federica Moret, Marco Luigetti, Vincenzo Di Stefano

**Affiliations:** ^1^ Department of Human Neuroscience Sapienza University of Rome Rome Italy; ^2^ IRCCS Istituto Delle Scienze Neurologiche Di Bologna Bologna Italy; ^3^ Dipartimento Di Neuroscienze, Organi Di Senso E Torace, UOC Neurologia Fondazione Policlinico Universitario Agostino Gemelli IRCCS, Largo Agostino Gemelli, 8 Rome Italy; ^4^ Department of Neurology, Mental Health and Sensory Organs (NESMOS), Faculty of Medicine and Psychology Sapienza University of Rome and UniCamillus‐Saint Camillus International University of Health Sciences Rome Italy; ^5^ AOU Careggi and Department of Neurosciences, Drug and Child Health University of Florence Florence Italy; ^6^ Neurology Unit, Department of Neuroscience University of Padua Padua Italy; ^7^ Neurology Unit Ospedale Santa Croce Di Fano Fano Italy; ^8^ Neurological Clinic, Department of Experimental and Clinical Medicine Marche Polytechnic University Ancona Italy; ^9^ Neurology Unit Great Metropolitan Hospital “Bianchi Melacrino Morelli” Reggio Calabria Italy; ^10^ Department of Neurology Infermi Hospital Rimini Italy; ^11^ Division of Cardiology University Hospital Paolo Giaccone Palermo Italy; ^12^ Department of Neuroscience, Imaging and Clinical Sciences “G. D'annunzio” University Chieti Italy; ^13^ Division of Neuroscience, Department of Neurology, Institute of Experimental Neurology San Raffaele Scientific Institute Milan Italy; ^14^ Department of Clinical and Experimental Sciences University of Brescia Brescia Italy; ^15^ NeMO‐Brescia Clinical Center for Neuromuscular Diseases Brescia Italy; ^16^ Asl TO4 Ciriè Italy; ^17^ IRCCS Ospedale Policlinico San Martino Genoa Italy; ^18^ Department of Clinical and Experimental Medicine University of Messina Messina Italy; ^19^ Neuromuscular Diseases Unit, Department of Systems Medicine Tor Vergata University of Rome Rome Italy; ^20^ Neuromuscular and Rare Disease Centre, Neurology Unit Sant'andrea Hospital Rome Italy; ^21^ Cardiology Unit, Cardiac Thoracic and Vascular Department IRCCS Azienda Ospedaliero‐Universitaria Di Bologna Bologna Italy; ^22^ Department of Neuroscience, Reproductive and Odontostomatological Science University of Naples ‘Federico II’ Naples Italy; ^23^ Neurology Unit, Department of Basic Medical Sciences, Neurosciences and Sense Organs University of Bari Aldo Moro Bari Italy; ^24^ S.C. Malattie Neurologiche Rare, Dipartimento Di Neuroscienze Cliniche Fondazione IRCCS Istituto Neurologico Carlo Besta Milan Italy; ^25^ Neurology Unit AV3, ASUR Marche Macerata Macerata Italy; ^26^ Unit of Neurology ASST Spedali Civili Brescia Italy; ^27^ Department of Neuroscience “Rita Levi Montalcini”, University of Turin Turin Italy; ^28^ IRCCS Istituto Auxologico Italiano Milan Italy; ^29^ Department of Neuroscience Università Cattolica Del Sacro Cuore Rome Italy; ^30^ Department of Neuroscience, Biomedicina E Movimento Università Di Verona Verona Italy; ^31^ Dipartimento Di Neurologia/Stroke Unit Ospedale Di Bolzano Bolzano Italy; ^32^ Neurology Unit, Department of Neurosciences University Hospital Santa Maria Della Misericordia Udine Italy; ^33^ Department of Internal Clinical, Anesthesiological and Cardiovascular Sciences “Sapienza” University of Rome Rome Italy; ^34^ Department of Biomedicine, Neuroscience and Advanced Diagnostics (BIND), University of Palermo Palermo Italy; ^35^ IRCCS Neuromed Pozzilli Italy; ^36^ Department of Medical and Surgical Sciences Magna Græcia University of Catanzaro Catanzaro Italy; ^37^ Regional Epilepsy Centre, Great Metropolitan “Bianchi‐Melacrino‐Morelli Hospital” Reggio Calabria Italy

**Keywords:** ATTRv, F64L mutation, NT‐proBNP, p. phe84leu, transthyretin amyloidosis

## Abstract

**Background:**

The F64L variant is among the most frequent TTR mutations in Italy, typically associated with a predominantly neurologic phenotype and limited cardiac involvement.

**Methods:**

Data from 181 ATTRv patients in the multicenter Patisiranitaly database treated with Patisiran since 2020 were analyzed. Neurologic impairment scores, Norfolk QoL‐DN, and cardiac parameters were compared between F64L (*n* = 56), V30M (*n* = 37), and non‐F64L (*n* = 125) patients at baseline and during follow‐up. Cluster analysis was applied to identify patient subgroups based on these variables.

**Results:**

F64L represented 30.9% of the cohort. Compared to non‐F64L patients, F64L patients had a higher prevalence of neurologic onset and neurologic phenotype, a thinner interventricular septum, and lower NT‐proBNP levels. Cluster analysis segregated patients into two distinct groups, predominantly reflecting F64L vs. non‐F64L status and corresponding neurologic severity. F64L patients showed milder cardiac involvement compared to V30M patients. Longitudinal repeated‐measures ANOVA showed stable clinical and instrumental measures.

**Conclusions:**

F64L is characterized by predominant neurologic involvement and milder cardiac involvement in this Patisiran‐treated cohort. Mutation‐specific diagnostic and follow‐up strategies are essential to capture its natural history and treatment response.

## 1 Introduction

Hereditary transthyretin‐mediated amyloidosis (ATTRv, “v” for “variant”) is a rare systemic disease characterized by amyloid deposition in multiple organs, particularly the peripheral nervous system and the heart, leading to heart failure and loss of unassisted ambulation [1]. In recent years, effective drugs have become available to stabilize disease progression, acting either as protein stabilizers [2, 3, 4] or as inhibitors of TTR synthesis [5, 6, 7]. Additional therapies aimed at deeply inhibiting hepatic TTR production or at TTR depletion are under investigation and may become available in the future [8, 9, 10, 11].

Despite therapeutic advances, several questions remain unresolved. The roles of extra‐hepatic TTR synthesis [12, 13, 14, 15], gender differences [16], genetic polymorphisms [17], susceptibility and modifier genes [18], and the clinical phenotypes associated with different mutations [19, 20] are still under investigation.

The epidemiology of individual TTR mutations varies widely. In North America, V122I is the most frequent mutation, particularly among African American individuals [21]. In endemic countries such as Portugal and Sweden, V30M is the most common [22]. Even within different regions of the same country, the prevalence of specific mutations can vary. In a recent study, V30M and F64L (also referred to as p. Phe84Leu, according to HGVS nomenclature) were reported as the most frequent mutations in Lazio [23]. Another report from this Italian ATTRv study group demonstrated that F64L is the most representative TTR mutation in Italy and may be considered an Italian‐specific variant, particularly in the central and southern regions, with only sporadic cases reported outside Italy, often in families of Italian origin [24]. This data was recently confirmed in other epidemiological studies [25, 26]. A distinctive feature of F64L is that the sensitivity of bone scintigraphy (DPD and HMDP) in detecting ATTR‐related cardiomyopathy is extremely low, suggesting that a different diagnostic algorithm may be required for these patients [27]. Low bone‐tracer affinity was previously observed in early‐onset V30M mutation too and attributed to the different types of fibrils (type B vs. type A) [28]. To our best knowledge, no paper has studied the type of fibrils in patients with the F64L mutation.

In this study, we focused our analysis on cardiac and neurologic characteristics of F64L in an Italian ATTRv database, comparing them with patients with non‐F64L mutations.

## 2 Methods

We analyzed data from the “Patisiranitaly” multicentric retrospective database, in which 29 Italian referral centers for ATTRv have been contributing data on Patisiran‐treated patients since February 2020 to January 2024. Sixty‐six patients switched to Vutrisiran during the period of data collection and were excluded from the database from the time of the switch.

Other inclusion criteria were: Signed informed consent; age ≥ 18 years; confirmed diagnosis of symptomatic hereditary ATTRv amyloidosis with length‐dependent sensorimotor neuropathy associated with a known amyloidogenic TTR variant; and absence of alternative causes of neuropathy (e.g., diabetes, inflammatory polyneuropathy, AL amyloidosis, chronic alcoholism, neuropathy due to anti‐MAG antibodies, or vitamin B12 deficiency). Eligible patients were required to be actively receiving patisiran in accordance with Italian regulations, without concomitant ATTRv therapies.

Exclusion criteria included lack of informed consent, patisiran discontinuation before 9 months of therapy, prior exposure to patisiran before 2020 (in clinical trials or compassionate use programs), concurrent participation in other pharmacological trials, active hepatitis B or C infection, and hepatic or renal failure. F64L patients were compared to V30M (also referred to as p. Val50Met, according to HGVS nomenclature) patients and to all non‐F64L ATTRv patients. All participants underwent cardiological and neurological examinations, assessment of clinical scales, and measurement of serum biomarkers at baseline (T0) and at 9‐month intervals (T1: 9 months; T2: 18 months; T3: 27 months; T4: 36 months), in accordance with the regulatory agency's monitoring requirements. The following parameters were collected: Genotype, age at symptom onset, type of onset (neurologic, cardiac, mixed, or other: Ocular, leptomeningeal, renal), isolated neuropathy or multisystem involvement (cardiac, neurologic, gastrointestinal, mixed‐phenotype, and other phenotype) at baseline, and age at diagnosis. Phenotypes were defined based on clinical and instrumental evidence of organ‐specific involvement. In particular, Gillmore's algorithm [29] (IVS thickness cut‐off: ≥ 12 mm) was used to confirm amyloid cardiomyopathy. In cases of non‐captant mutations with a hypertrophic phenotype, cardiac MRI or endomyocardial biopsy was required to exclude cardiac involvement. Nerve conduction studies were performed to assess amyloid polyneuropathy, while tissue biopsy was used to evaluate involvement of other organs [30]. Clinical assessments included: Familial Amyloid Polyneuropathy (FAP) stage, NYHA functional class at baseline, Neuropathy Impairment Score (NIS), Norfolk QoL‐DN questionnaire, Karnofsky Performance Status (KPS), 6‐min walking test (6MWT), modified BMI (mBMI), and Compound Autonomic Dysfunction Test (CADT). Instrumental investigations included transthoracic echocardiography to measure interventricular septum thickness (IVS thickness), nerve conduction studies with neurophysiological score, and serum NT‐proBNP as a cardiac biomarker.

The study was approved by the Ethical Committee of Palermo (June 26, 2023, V n.6/2023) and has therefore been performed in accordance with the ethical standards laid down in the 1964 Declaration of Helsinki and its later amendments. All participants signed informed consent for data publication.

### 2.1 Statistical Analysis

Demographic data are presented as median (Interquartile Range‐IQR). Continuous variables were compared using the Mann–Whitney U test or Student's *t*‐test, according to data distribution, and associations between continuous variables and dichotomous factors were additionally assessed using the point‐biserial correlation. Associations between genotype (F64L vs. non‐F64L) and clinical phenotype were evaluated using Pearson's chi‐square test, with standardized residuals analyzed to identify which categories contributed most to the association. Longitudinal assessments were evaluated across all the available timepoints using repeated‐measures ANOVA (RM‐ANOVA) or the Friedman test as appropriate.

Two‐Step Cluster analysis was performed on 135 patients using phenotype, NYHA class, NIS, IVS, and Norfolk QoL‐DN. Variables were selected according to the clinical relevance and the significance of the Pearson's chi‐square test between F64L and other groups. F64L status was included as a categorical variable. The number of clusters was determined automatically using Schwarz's BIC, a model selection criterion that balances goodness‐of‐fit and model complexity by introducing a penalty term for the number of parameters; lower values indicate a better model. This approach was used to identify patient subgroups based on the simultaneous evaluation of multiple clinical and functional variables, providing a multidimensional perspective on phenotypic patterns beyond univariate analyses and confirming the distinct profile of F64L patients. Cluster quality, centroids, and the association between F64L and cluster membership (chi‐square test) were examined.

A multivariate analysis of variance (MANOVA) was performed to assess whether the same scalar variables considered in the cluster analysis (NIS, IVS, and Norfolk QoL‐DN) differed between patients with and without the F64L variant. When the MANOVA was significant, univariate ANOVAs were conducted for each dependent variable.

Multiple linear regression analysis was performed to assess the independent association of the F64L variant with IVS, controlling for age at diagnosis and sex. Collinearity diagnostics were evaluated using Tolerance and Variance Inflation Factor (VIF), confirming low multicollinearity among predictors.

Statistical significance was set as *p* < 0.05. All the analyses were performed with SPSS 25.0.

## 3 Results

**FIGURE 1:**
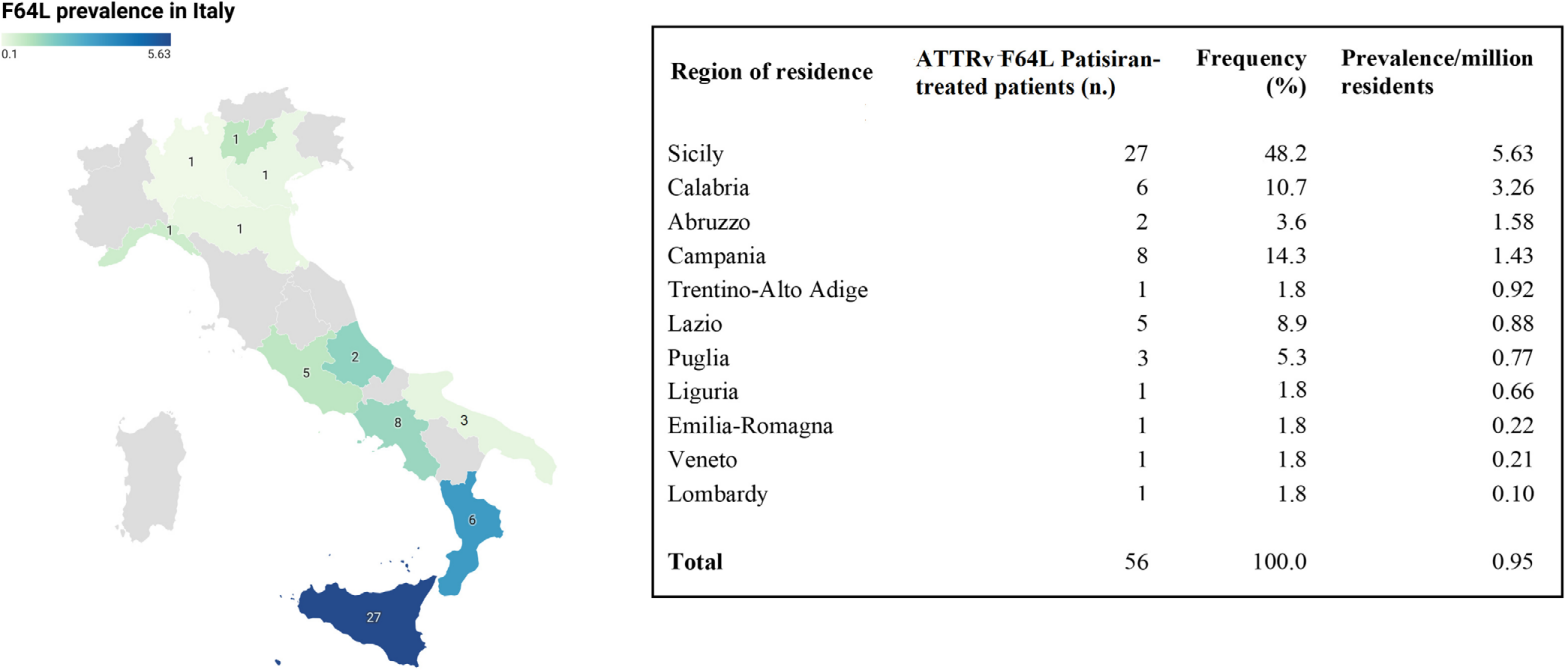
Regional distribution of ATTRv F64L Patisiran‐treated patients in Italy. Left panel: Map of Italy with regions color‐coded according to F64L mutation prevalence per million inhabitants. Darker shades indicate higher prevalence. Population data are from ISTAT (2023). Right panel: Table reporting for each region, the number of F64L ATTRv Patisiran‐treated patients, percentage of the total cohort of patients, and prevalence of Patisiran‐treated patients per million inhabitants.

Retrospectively collected data from 181 ATTRv patients treated with Patisiran wereanalyzed. Among them, 56 (30.9%) carried the F64L variant, making it the most represented mutation. Other mutations included I68L (21.0%), V30M (20.4%), E89Q (8.3%), V122I (3.9%), and others (Table S1 for further details). The prevalence of ATTRv F64L Patisiran‐treated patients across different Italian regions is shown in Figure 1.

Demographic comparisons (Table 1) revealed a male prevalence of 66.4% in F64L vs. 75.7% in V30M patients and 73.2% in non‐F64L patients (*p* > 0.05). Only 3 of 37 V30M patients were considered early onset, considering the age of onset. Clinical phenotypes in F64L vs. V30M vs. non‐F64L were classified as predominantly cardiac, predominantly neurologic, predominantly gastroenterologic, and mixed. Pearson's chi‐square test indicated that phenotypes were differently distributed between F64L and non‐F64L (χ^2^ = 34.215, df = 4, *p* < 0.001). Standardized residuals showed neurologic phenotypes were more frequent in F64L (+2.3) and cardiac phenotypes less frequent (−2.2), whereas mixed and gastroenterologic phenotypes showed no significant deviations. No significant difference was found in clinical phenotype between F64L and V30M patients.

**TABLE 1:** Demographic and clinical baseline characteristics of patients with F64L, V30M, and all non‐F64L mutations. data are presented as median (interquartile range) for continuous variables and percentage for categorical variables. abbreviations: MBMI, modified Body Mass Index; NIS, Neuropathy Impairment Score; KPS, Karnofsky Performance Status; QoL‐DN, Norfolk Quality of Life–Diabetic Neuropathy; CADT, Compound Autonomic Dysfunction Test; 6MWT, Six‐Minute Walk Test; IVS, interventricular septal thickness; NYHA, New York Heart Association functional class; m, meters, mm, millimeters. *p*‐values refer to comparisons between F64L and non‐F64L groups.

	F64L (*n* = 56)	V30M (*n* = 37)	All non‐F64L (*n* = 125)
Age at diagnosis, years	68 (52–74)	68 (63.2–72.7)	66 (51–74)
Sex (male), %	73.2	75.7	66.4
Phenotype at onset, %	**		
Cardiac	8.9^a^	8.1	37.6
Neurologic	80.4^a^	83.8	52.0
Mixed phenotype	3.6	0.0	4.8
Gastroenterologic	5.4	5.4	4.8
Other/*n*.s.	1.8	2.7	0.8
FAP stage, %			
I	69.6	56.8	72.8
II	30.4	43.2	27.2
NYHA functional class, %	**		
I	76.8^a^	56.8	45.6
II	14.3^a^	27.0	37.6
III	7.1	16.2	16.0
IV	1.8	0	0.8
Perugini score, %	**; ****		
0	87.2^a^	15.0	8.6
1	12.8	10.0	8.6
2	0.0^a^	5.0	38.7
3	0.0^a^	70.0	44.1
mBMI	901 (842–1,046)	925,9 (828.1–1,089)	929.4 (813–1131.5)
NIS	48 (28.7–73.4)**	46 (21.5–68.5)	25 (12–48.5)
KPS	70 (60–87.5)**;***	80 (70–90)	80 (70–90)
Norfolk QoL‐DN	56 (35–74)**	50 (31–67)	36 (17–60)
CADT	16 (12–18)	16 (12–18)	16 (13–20)
‐ Male	18 (16–20)*	16 (12–18)	18 (16–20)
‐ Female	14 (12–18.75)	16 (12–18)	14 (12–18.25)
6MWT, m	269 (212–317)	325 (253–410)	300 (170–380)
IVS thickness, mm	12 (10.5–13)**;****	14.5 (11.7–18.2)	16 (13–18.4)
NT‐proBNP, pg/mL	163 (64–811)**;****	600 (239–1,094)	703 (224–1,855)

^*^ *p* < 0.05 in F64 vs All non‐F64L.

^**^ *p* < 0.01 in F64 vs All non‐F64L.

^***^ *p* < 0.05 in F64 vs V30M.

^****^ *p* < 0.01 in F64 vs V30M; All non‐F64L.

^a^ Standardized residual| > 1.96 for F64L vs All non‐F64L.

Disease onset type was differently distributed in F64L compared to non‐F64L patients (χ^2^ = 16.68, df = 4, *p* = 0.002). Cardiac onset was less frequent in F64L (−2.8) compared with non‐F64L (+1.9); moreover, neurologic onset was more frequent in F64L (+1.9) and less common in non‐F64L (−1.3), although these last differences did not reach statistical significance. No significant difference was found in disease onset between F64L and V30M patients.

NYHA class was differently distributed with F64L status compared to all non‐F64L patients (χ^2^ = 16,35, df = 3, *p* < 0.01). F64L patients were overrepresented in class I (+2.2) and underrepresented in classes II (−2.2) and III (−1.3, n.s.). Non‐F64L patients showed the opposite pattern. No difference was found in NYHA class between F64L and V30M patients.

Analysis of Perugini scores revealed that F64L patients mostly had scores of 0 or 1, with no scores of 2 or 3, as expected. In non‐F64L patients, scores of 2 (38.7% vs. 0%, residual = 2.1) and 3 (44.1% vs. 0%, residual = 2.3) were overrepresented compared to F64L, with fewer scores of 0 (8.6% vs. 87.2%, residual = −4.0). In V30M patients, scores of 3 (70% vs. 0%, residual = 4.2) were overrepresented compared to F64L patients, with fewer scores of 0 (15% vs. 87.2, residual = −2.7). The distribution of Perugini scores was significantly different for F64L compared to both V30M (χ^2^ = 40.32, df = 3, *p* < 0.001) and non‐F64L patients (χ^2^ = 86.11, df = 3, *p* < 0.001).

At baseline, F64L patients showed higher NIS scores (*p* < 0.01) with a significant association (rpb, *p* < 0.01), higher Norfolk QoL scores (*p* < 0.01) with a significant association (rpb, *p* < 0.01), and lower KPS (*p* < 0.01) with a significant association (rpb, *p* < 0.01), compared to non‐F64L patients. IVS thickness (*p* < 0.01; rpb, *p* < 0.01) and NT‐proBNP (*p* < 0.01; rpb, *p* < 0.05) were also lower in F64L patients. No significant differences were observed in age at onset or diagnosis, weight, mBMI, CADT, or 6MWT. When stratified by sex, CADT was reduced in F64L compared to non‐F64L males (*p* < 0.05; rpb, *p* < 0.05).

When compared to V30M, F64L showed lower KPS (*p* < 0.05; rpb, *p* < 0.05), IVS thickness (*p* < 0.01; rpb, *p* < 0.01), and NT‐ProBNP (*p* < 0.01; rpb, *p* > 0.05).

**FIGURE 2:**
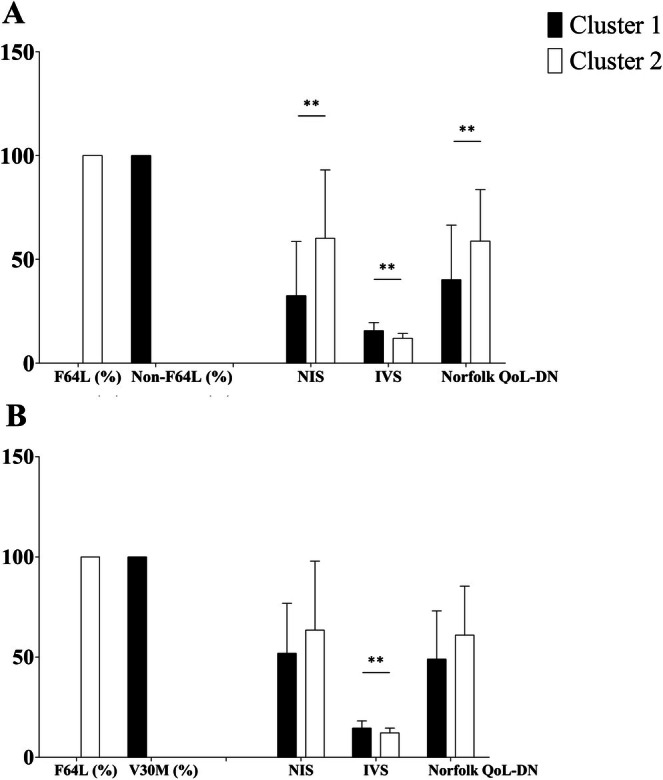
Cluster analysis of patients with F64L and non‐F64L variants (A) and F64L and V30M variants (B). Patients were stratified into two clusters based on clinical and instrumental features. The distribution of genetic variants (%) is reported on the left, followed by clinical and instrumental parameters: NIS (Neuropathy Impairment Score), IVS (interventricular septum thickness, mm), and Norfolk QoL‐DN (Norfolk Quality of Life–Diabetic Neuropathy questionnaire score). Data are presented as mean ± standard deviation (SD). In panel A, Cluster 1 includes patients without the F64L mutation, whereas Cluster 2 includes patients with the F64L mutation. In panel B, Cluster 1 includes patients with the V30M mutation, whereas Cluster 2 includes patients with the F64L mutation. **p < 0.01.

Two clusters were identified at the cluster analysis of F64L vs. non‐F64L patients, based on five selected variables (Figure 2A): Phenotype, NYHA class, NIS, IVS, and Norfolk QoL‐DN (Figure 2). Variables were selected according to the clinical relevance and the significance of the Pearson's chi‐square test. Cluster 1 comprised 97 patients (71.9% of the analyzed sample) and Cluster 2 included 38 patients (28.1% of the analyzed sample). Cluster quality was rated as sufficient. Cluster centroids showed that patients in Cluster 1 had lower NIS (32.5 ± 26.1 vs. 60.2 ± 32.9, *p* < 0.01), higher IVS thickness (15.6 ± 4.0 mm vs. 12.0 ± 2.3 mm, *p* < 0.01), and lower Norfolk QoL‐DN scores (40.2 ± 26.3 vs. 58.8 ± 24.8, p < 0.01) compared to Cluster 2. Notably, F64L patients were exclusively clustered in Cluster 2 (*n* = 38; 100%), whereas non‐F64L patients were exclusively clustered in Cluster 1 (*n* = 97; 100%). This distribution was highly significant (χ^2^ = 135.0, df = 1, *p* < 0.001). Cluster 2 highly contributed to the neurologic phenotype (63.6% of patients), whereas Cluster 1 mainly contributed to the cardiac phenotype (89.5% of patients). NYHA class also differed between clusters, with Cluster 2 mainly contributing to class 1 (58.8% of NYHA I patients) and Cluster 1 more represented in classes 2–3 (86.7% and 85% of NYHA II and III patients).

Once geographical regions were grouped into Northern, Central, and Southern Italy (including Islands) according to standard national classifications, Cluster 2 was significantly more represented in Southern Italy (standardized residual 3.8, χ^2^ = 35.7, df = 2, *p* < 0.001).

Although univariate analyses already highlighted significant differences between F64L and non‐F64L patients, cluster analysis provides a complementary, multidimensional perspective. By simultaneously considering NIS, IVS, Norfolk QoL‐DN, NYHA class, and phenotype, this analysis confirms that F64L Patisiran‐treated patients consistently form a distinct cluster characterized by greater neurologic involvement and lower cardiac involvement. Thus, cluster analysis reinforces and visualizes the overall phenotypic pattern in a clinically meaningful way.

The MANOVA confirmed a significant multivariate effect of F64L status on the combination of IVS thickness, NIS, and Norfolk QoL‐DN. (Pillai's Trace = 0.282, F(3, 131) = 17.124, *p* < 0.001, partial η^2^ = 0.282). Follow‐up univariate ANOVAs showed significant differences for IVS thickness (F = 27.196, *p* < 0.001, partial η^2^ = 0.170), NIS (F = 26.421, *p* < 0.001, partial η^2^ = 0.166), and Norfolk QoL‐DN (F = 14.130, *p* < 0.001, partial η^2^ = 0.096).

Two clusters were identified at the cluster analysis of F64L vs. V30M patients, based on the same previously assessed variables (Figure 2B). Cluster 1 comprised 20 patients (38.5% of the analyzed sample) and Cluster 2 included 32 patients (61.5% of the analyzed sample). Cluster 1 consisted exclusively of V30M patients, whereas Cluster 2 included only F64L patients. The overall cluster quality was rated as sufficient. Compared with Cluster 2, Cluster 1 patients showed less severe, even if not significant, neurological impairment (NIS 51.9 ± 24.9 vs. 63.5 ± 34.4; Norfolk QoL‐DN 49.0 ± 24.1 vs. 61.0 ± 24.4, *p* > 0.05 for both comparisons) and significantly greater cardiac involvement (IVS 14.6 ± 3.6 vs. 12.2 ± 2.4, *p* < 0.05). Phenotypically, cardiac presentation was dominated by Cluster 1 (62.5%), whereas neurologic (87.5%) and gastroenterologic (75.0%) presentations were mainly represented by Cluster 2, and mixed (66.7%) phenotype by Cluster 2. The association between cluster membership and clinical phenotype was highly significant (Pearson χ^2^ = 58.0, df = 2, *p* < 0.001).

Once geographical regions were grouped into Northern, Central, and Southern Italy (including Islands) according to standard national classifications, Cluster 1 was significantly more represented in the Center of Italy (standardized residual 2.1, χ^2^ = 10.7, df = 2, *p* < 0.01).

These findings indicate that V30M patients in Cluster 1 represent a distinct clinical subgroup with more pronounced cardiac involvement than F64L patients in the other clusters.

The MANOVA confirmed a significant multivariate effect of genotype (F64L vs. V30M) on the combination of IVS thickness, NIS, and Norfolk QoL‐DN (Pillai's Trace = 0.213, F(3, 54) = 4.879, *p* = 0.004, partial η^2^ = 0.213), indicating that the groups differ on the combined measures. Follow‐up univariate ANOVAs showed that IVS differed significantly between genotypes (F = 11.051, *p* < 0.01, partial η^2^ = 0.165), whereas NIS (F = 0.973, *p* > 0.05) and Norfolk QoL‐DN (F = 2.077, *p* > 0.05) did not reach statistical significance.

**FIGURE 3:**
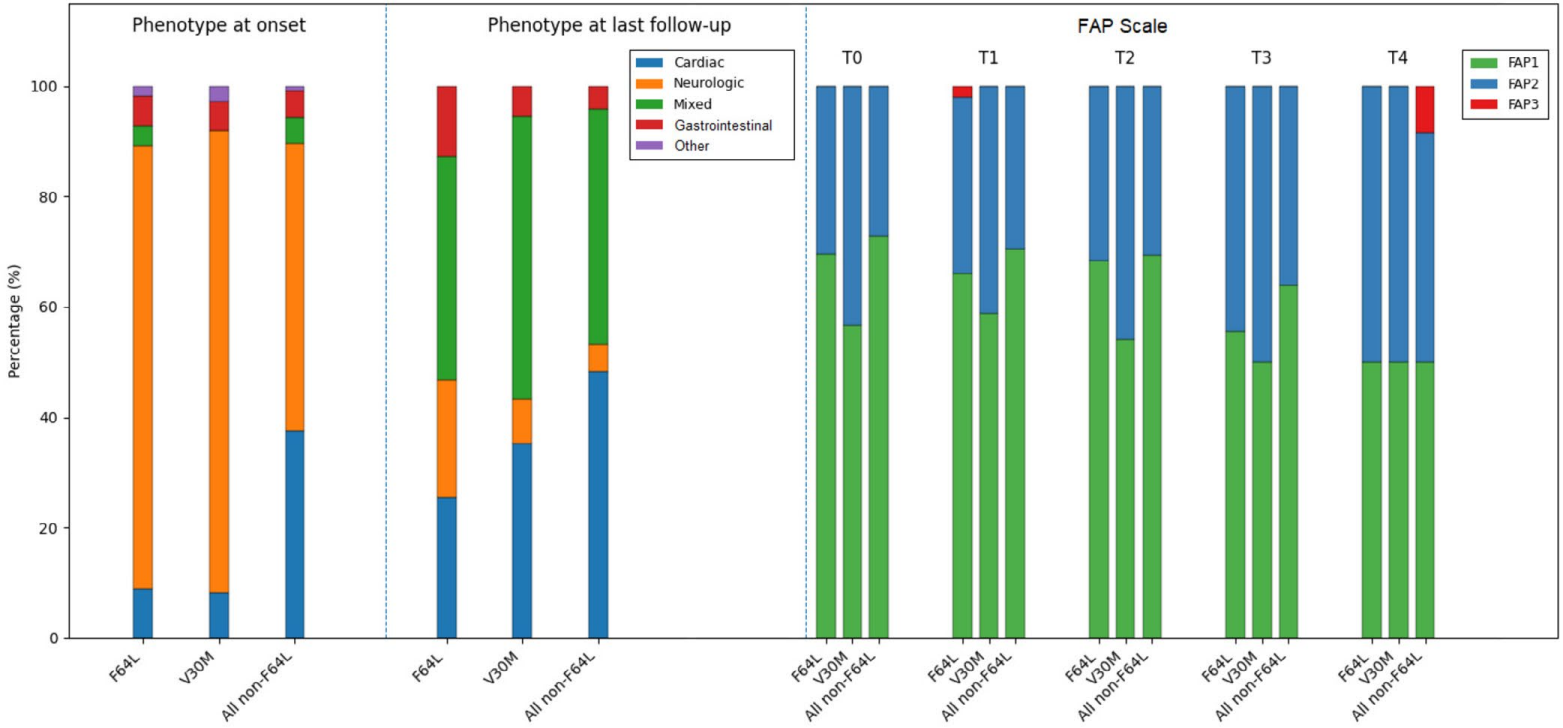
Phenotypic distribution and disease severity progression across TTR genotypes. Stacked bar charts illustrate the distribution of clinical phenotypes at disease onset (left panel) and at last follow‐up (middle panel), as well as the progression of disease severity according to FAP stage over time (right panel), stratified by genotype (F64L, V30M, and all non‐F64L variants). All values are expressed as percentages within each genotype group at each time point.

Longitudinal data are reported in Table S2; phenotypes at onset and at the last follow‐up, together with FAP stages across different time points, are shown in Figure 3.

RM‐ANOVA indicated no significant changes from T0 to T4 between F64L vs. V30M nor vs. non‐F64L patients for NIS, KPS, Norfolk QoL, CADT (even when stratified for sex), IVS thickness, or NT‐proBNP.

RM‐ANOVA with F64L vs. non‐F64L as the between‐subject factor showed a significant difference in the change of 6MWT from baseline to the first follow‐up at 9 months between the two groups, with no differences in the further timepoints. Specifically, non‐F64L patients improved their 6MWT from a mean of 284 m at baseline to 306 m at 9 months, whereas F64L patients showed a decrease from 288 m to 282 m, indicating a statistically significant group‐by‐time interaction for this timepoint (*p* < 0.01). No differences were found with V30M as a between‐subject factor.

In a multiple linear regression model including age and sex as covariates, the F64L variant remained independently associated with IVS thickness (B = −4.44, β = −0.50, *p* < 0.001), with low collinearity (Tolerance = 0.96, VIF = 1.04).

## 4 Discussion

In this cohort of 181 ATTRv patients treated with Patisiran, 56 (30.9%) carried the F64L variant, making it the most common mutation in this representative cohort of Italian Patisiran‐treated patients. To our best knowledge, this is the most representative cohort of ATTRv F64L Patisiran‐treated patients in the literature. Our data are consistent with those of Russo et al. [26], who reported 58 F64L ATTRv patients from the Italian registry, and Fumagalli et al. [25], who reported 22% of F64L prevalence among ATTRv patients in Italy. In both studies, in contrast to the present one, all patients with ATTRv were included, regardless of therapy. The age at diagnosis was non‐significantly higher in F64L patients and consistent with previous data from the THAOS registry [31].

F64L Patisiran‐treated patients showed a higher prevalence in southern Italy, as previously reported in the ATTRv population [25, 26], with Sicily and Calabria exhibiting more than twice the prevalence observed in other regions. Sporadic cases were also identified in central and northern regions, likely reflecting intra‐country migration.

F64L predominantly showed a neurologic phenotype, as indicated by higher rates of predominant neurologic onset (80.4% vs. 52.0%) and lower prevalence of cardiac phenotypes (8.9% vs. 37.6%) compared with non‐F64L patients. No differences were found between the clinical phenotype distribution of F64L and V30M patients.

These findings were supported by analyses of onset phenotype, NYHA functional class, and cardiac involvement assessed by Perugini scores, echographic IVS thickness, and NT‐proBNP. F64L patients were larger than non‐F64L patients in lower NYHA classes, showed reduced cardiac uptake on bone scintigraphy, and had lower IVS thickness and NT‐proBNP levels, consistent with milder cardiac involvement. Some F64L patients may be classified as predominantly neurologic, primarily due to negative bone scintigraphy, a well‐known characteristic of this variant [27]. All the F64L patients in this cohort were considered to have a cardiac/mixed phenotype (7/7) at onset according to the results of CMR, given the Perugini score < 2. The negative result of cardiac scintigraphy may reflect a low affinity for bone tracers rather than a true absence of amyloid infiltration, eventually related to the type of fibrils, as previously hypothesized for early‐onset V30M patients [28]; further studies are requested to deepen the relationship between F64L fibrils and bone‐tracer affinity. In endemic countries with a high prevalence of non‐captant early‐onset V30M mutation, the presence of the mutation + consistent symptoms and typical echo/CMR findings was considered sufficient for the diagnosis of cardiac involvement [32]. Indeed, in previous studies, amyloid deposition was detected in 75% of F64L patients with negative scintigraphy who underwent cardiac MRI, and in 1/1 patient with negative scintigraphy who underwent endomyocardial biopsy [27], thus suggesting an adaptation of the flow chart proposed by Gilmore et al. [29].

Nevertheless, NYHA class, IVS thickness, and NT‐proBNP data confirm milder cardiac involvement in F64L compared to all non‐F64L patients in this cohort. F64L patients showed a reduced IVS thickness and NT‐proBNP when compared to V30M too, suggesting milder cardiac involvement, even relative to the most prevalent variant in international registries.

Our analysis demonstrates that the F64L variant is independently associated with reduced IVS thickness, even after adjusting for age and sex. The low collinearity observed between F64L, age, and sex supports the notion that this genetic factor influences cardiac impairment independently of these demographic variables. Cardiac denervation rather than amyloid deposition may underlie some cardiac manifestations in F64L, as suggested by reports of ^123^I‐mIBG scintigraphy showing sympathetic denervation despite negative bone scans in a previous study [33]. Systematic studies using ^123^I‐mIBG or cardiac MRI in F64L patients are warranted to better quantify cardiac involvement in this specific variant.

Neurologic burden in F64L patients was reflected in higher NIS and Norfolk QoL scores and lower KPS compared to non‐F64L patients. This involvement seems to be similar to the V30M mutation. The cluster analysis demonstrates in a multidimensional way that the F64L variant defines a phenotypically distinct subgroup among this cohort of patients. Patients with F64L were exclusively grouped in a cluster characterized by higher neuropathic burden (higher NIS, worse Norfolk scores) and milder cardiac involvement (lower IVS thickness), consistent with previous observations of predominantly neurologic expression and negative bone scintigraphy in this mutation. The strong association between F64L status and cluster membership underscores the influence of genotype on this clinical phenotype. Even when compared with V30M, F64L patients were clustered in a group characterized by milder cardiac involvement and more severe neurological impairment. F64L clusters were more represented in Southern Italy. These results highlight the utility of cluster‐based approaches to identify mutation‐specific phenotypic patterns, which may inform tailored diagnostic algorithms and patient management strategies.

Finally, F64L males even showed a higher disautonomic impairment compared to non‐F64L patients, as evidenced by a lower CADT score. CADT is a disautonomic scale with different scale ranges for males and females, given an additional item for sexual dysfunction in males. Reduced values of CADT in F64L males can suggest a higher small‐fiber impairment in this genotype.

Longitudinal analyses showed stability over time in NIS, KPS, Norfolk QoL, CADT (even when stratified for sex), IVS thickness, and NT‐proBNP, regardless of the genotype. Post hoc analysis of the 6MWT revealed a subtle walking disability in F64L patients at 9 compared to baseline, despite limited cardiac involvement. These findings suggest that the decline at this early time point after therapy onset is primarily driven by neurological impairment, which is subsequently recovered in the later available follow‐up assessments. The absence of a control group precludes definitive conclusions, and thus these findings should be considered speculative.

Limitations of this study include a retrospective design, potential selection bias toward patients treated with Patisiran, and limited data on small‐fiber involvement. In particular, siRNA therapy is nowadays approved only in the presence of polyneuropathy, with or without cardiomyopathy. Nonetheless, within this cohort of patients with polyneuropathy, the F64L mutation appears to be less frequently associated with cardiomyopathic involvement and with more significant neurologic involvement compared to the other TTR mutations of the cohort. The prevalence of cardiomyopathy may be underestimated in this cohort due to the inclusion of patients enrolled in clinical trials with cardiologic endpoints and the concomitant use of TTR stabilizers at the time of data collection. However, these factors are expected to be independent of the specific TTR mutation; therefore, inter‐group differences are unlikely to be significantly affected by this potential bias.

Another limitation of the present study is the restricted availability of data on small‐fiber involvement in F64L compared with non‐F64L patients. In a single‐center experience, dysautonomia has been reported as moderate in patients with this mutation [34]. Although the CADT score was reduced in F64L males, future studies should include a broader panel of assessments (e.g., COMPASS‐31, quantitative sensory testing, epidermal nerve fiber density) to more accurately characterize small‐fiber involvement in F64L patients.

## 5 Conclusion

In conclusion, the F64L variant emerges as a distinct ATTRv mutation with milder cardiac involvement, even when compared to V30M, and a predominantly neurologic phenotype in this cohort of Patisiran‐treated ATTRv patients. These findings highlight the need for tailored diagnostic algorithms in F64L patients. Future studies integrating advanced autonomic and small‐fiber assessments, as well as cardiac MRI, are warranted to refine the phenotypic characterization and improve clinical management of F64L‐related ATTRv amyloidosis.

## Author Contributions

**Marco Ceccanti:** conceptualization, investigation, funding acquisition, methodology, validation, writing – review and editing, software, formal analysis, project administration, data curation, writing – original draft. **Pietro Guaraldi:** investigation. **Marco Burattini:** investigation. **Marco Currò Dossi:** investigation. **Micol Gianoli:** investigation. **Sabrina Gasverde:** investigation. **Vittoria Cianci:** investigation. **Daniela Di Lisi:** investigation. **Alessandro Barilaro:** investigation. **Adele Ratti:** investigation. **Chiara Briani:** investigation. **Antonio Di Muzio:** investigation. **Massimiliano Filosto:** investigation. **Giulia Carlini:** investigation. **Chiara Gemelli:** investigation. **Luca Gentile:** investigation. **Angela Romano:** investigation. **Mariangela Goglia:** investigation. **Giovanni Antonini:** investigation. **Simone Longhi:** investigation. **Sofia Maria Augello:** investigation. **Luca Leonardi:** investigation. **Giuseppina Novo:** investigation. **Davide Pareyson:** data curation, writing – review and editing. **Fiore Manganelli:** investigation. **Cristina Petrelli:** investigation. **Antonio Lotti:** investigation. **Luigi Sellitti:** investigation. **Silvia Fenu:** investigation. **Massimo Russo:** investigation. **Loris Poli:** investigation. **Anna Mazzeo:** investigation. **Giammarco Milella:** investigation. **Giovanni Palumbo:** investigation. **Alessandro Salvalaggio:** investigation. **Luca Guglielmo Pradotto:** investigation. **Maria Ausilia Sciarrone:** investigation. **Maurizio Inghilleri:** methodology, writing – review and editing, supervision. **Mariagiovanna Castiglia:** investigation. **Matteo Tagliapietra:** investigation. **Mara Turri:** investigation. **Francesca Vitali:** investigation. **Roberto D'Angelo:** investigation. **Lorenzo Verriello:** investigation. **Stefano Tozza:** investigation. **Nicasio Rini:** investigation. **Chiara Cambieri:** data curation, investigation. **Laura Libonati:** investigation, data curation. **Filippo Brighina:** investigation. **Cristina Chimenti:** investigation. **Federica Moret:** data curation, investigation. **Marco Luigetti:** writing – review and editing, methodology, investigation, writing – original draft. **Vincenzo Di Stefano:** methodology, writing – review and editing, investigation, writing – original draft. **Domenico Abelardo:** data curation, writing – review and editing.

## Funding

This study was supported by this manuscript did not receive any funding.

## Conflicts of Interest

The authors declare no conflicts of interest.

## Supporting information


**Table S1:** Other *TTR* variants (*n* = 30).

**Table S2:** Longitudinal data of F64L, V30M, and all non‐F64L cohorts. Data are presented as median (interquartile range). Abbreviations: mBMI, modified Body Mass Index; NIS, Neuropathy Impairment Score; KPS, Karnofsky Performance Status; QoL‐DN, Norfolk Quality of Life–Diabetic Neuropathy; CADT, Compound Autonomic Dysfunction Test; 6MWT, Six‐Minute Walk Test; IVS, interventricular septal thickness; NYHA, New York Heart Association functional class; m, meters, mm, millimeters.

## Data Availability

The data that support the findings of this study are available from the corresponding author upon reasonable request.
