# Supplementary material for: Deep Phenotyping of F64L Mutation in a Multicentric Cohort of Patisiran‐Treated Hereditary Transthyretin Amyloidosis Patients (Patisiranitaly)
Source: Eur J Neurol. 2026 Jun 1;33(6):e70657. doi: 10.1111/ene.70657 (PMC13240185; doi:10.1111/ene.70657)
Supplement: Supplementary file 1 — Table S1: Other TTR variants (n = 30). [file ENE-33-e70657-s002.docx]

**Supplementary Table 1.** Other *TTR* variants (*n* = 30).

| TTR variant | Count (%) |
| --- | --- |
| p.Glu54Gln | 4 (2.2%) |
| p.Ala36Pro | 4 (2.2%) |
| p.His90Asn | 2 (1.1%) |
| p.Phe64Ile | 2 (1.1%) |
| p.Tyr78Phe | 2 (1.1%) |
| p.Val94Leu | 2 (1.1%) |
| p.Ala109Ser | 2 (1.1%) |
| p.Ala120Thr | 1 (0.6%) |
| p.Ala45Thr | 1 (0.6%) |
| p.Arg34Thr | 1 (0.6%) |
| p.Glu92Lys | 1 (0.6%) |
| p.Glu51Gln | 1 (0.6%) |
| p.Glu54Lys | 1 (0.6%) |
| p.Phe33Val | 1 (0.6%) |
| p.Pro24Ser | 1 (0.6%) |
| p.Val30Ala | 1 (0.6%) |
| p.Val12Ala | 1 (0.6%) |
| p.Val122Ile/p. Phe64Leu | 1 (0.6%) |
| p.Val30Met/p.Val122Ile | 1 (0.6%) |
