# Supplementary material for: Deep Phenotyping of F64L Mutation in a Multicentric Cohort of Patisiran‐Treated Hereditary Transthyretin Amyloidosis Patients (Patisiranitaly)
Source: Eur J Neurol. 2026 Jun 1;33(6):e70657. doi: 10.1111/ene.70657 (PMC13240185; doi:10.1111/ene.70657)
Supplement: Supplementary file 2 — Table S2: Longitudinal data of F64L, V30M, and all non‐F64L cohorts. Data are presented as median (interquartile range). Abbreviations: mBMI, modified Body Mass Index; NIS, Neuropathy Impairment Score; KPS, Karnofsky Performance Status; QoL‐DN, Norfolk Quality of Life–Diabetic Neuropathy; CADT, Compound Autonomic Dysfunction Test; 6MWT, Six‐Minute Walk Test; IVS, interventricular septal thickness; NYHA, New York Heart Association functional class; m, meters, mm, millimeters. [file ENE-33-e70657-s001.docx]

|  | F64L | V30M | All-nonF64L |
| --- | --- | --- | --- |
| NT-proBNP T1 | 162 (60–591) | 518 (160–1041) | 549 (147–1575) |
| NT-proBNP T2 | 112 (67–592) | 532 (210–1105) | 676 (221–1897) |
| NT-proBNP T3 | 162 (78–644) | 410 (235–955) | 474 (232–1634) |
| NT-proBNP T4 | 307 (98–1230) | 330 (238–813) | 330 (232–2697) |
| IVS T1 | 12.0 (10.0–13.0) | 14.5 (11.8–18.3) | 16.0 (13.0–18.0) |
| IVS T2 | 12.0 (11.0–13.3) | 16.0 (12.3–19.0) | 16.0 (13.3–18.5) |
| IVS T3 | 12.0 (11.0–12.3) | 14.5 (11.5–19.3) | 16.0 (13.0–17.8) |
| IVS T4 | 13.5 (12.3–14.8) | 13.0 (10.0–14.9) | 15.0 (12.3–18.3) |
| 6MWT T1 | 253 (173–324) | 335 (259–392) | 320 (236–390) |
| 6MWT T2 | 259 (205–305) | 325 (169–372) | 300 (206–345) |
| 6MWT T3 | 170 (143–241) | 300 (165–383) | 250 (130–380) |
| 6MWT T4 | 196 (196–196) | 335 (190–360) | 290 (355.0–390) |
| CADT T1 | 16.0 (14.0–18.0) | 14.0 (12.0–16.0) | 16.0 (12.5–19.5) |
| CADT T2 | 16.0 (14.0–19.0) | 14.0 (12.0–16.0) | 16.0 (13.0–20.0) |
| CADT T3 | 16.0 (15.0–16.0) | 14.0 (12.0–16.0) | 15.5 (14.0–18.8) |
| CADT T4 | 15.5 (14.3–16.8) | 16.0 (13.0–17.5) | 14.0 (12.0–17.0) |
| Norfolk QoL-DN T1 | 53.0 (30.0–75.0) | 45.0 (25.0–70.5) | 35.0 (18.3–55.8) |
| Norfolk QoL-DN T2 | 51.0 (17.0–69.5) | 48.5 (22.3–80.0) | 32.5 (17.8–61.3) |
| Norfolk QoL-DN T3 | 54.5 (37.5–72.5) | 38.0 (29.0–82.0) | 32.0 (19.3–72.5) |
| Norfolk QoL-DN T4 | 64.0 (24.8–77.0) | 58.0 (22.5–99.5) | 36.0 (20.0–91.0) |
| KPS T1 | 70.0 (60.0–80.0) | 80.0 (65.0–90.0) | 80.0 (70.0–90.0) |
| KPS T2 | 70.0 (70.0–80.0) | 80.0 (60.0–85.0) | 80.0 (60.0–90.0) |
| KPS T3 | 70.0 (60.0–80.0) | 70.0 (60.0–100.0) | 80.0 (60.0–90.0) |
| KPS T4 | 65.0 (60.0–70.0) | 60.0 (45.0–75.0) | 60.0 (50.0–82.5) |
| NIS T1 | 49.0 (26.3–74.3) | 52.0 (23.3–70.3) | 25.0 (14.0–50.8) |
| NIS T2 | 47.3 (18.5–69.8) | 60.3 (37.8–90.5) | 22.5 (13.0–60.4) |
| NIS T3 | 53.0 (24.0–76.5) | 62.5 (27.0–76.5) | 28.5 (12.8–64.0) |
| NIS T4 | 69.0 (44.0–88.0) | 64.0 (28.0–78.5) | 63.5 (16.3–90.5) |
| mBMI T1 | 940.0 (807.7–1047.0) | 960.5 (854.1–1138.5) | 927.7 (821.8–1042.3) |
| mBMI T2 | 891.0 (782.1–1126.0) | 887.5 (773.3–1023.3) | 927.0 (821.3–1062.0) |
| mBMI T3 | 903.0 (828.5–1091.5) | 924.2 (785.6–1058.0) | 925.3 (820.9–1072.5) |
| mBMI T4 | 1048.0 (710.3–1078.3) | 901.5 (797.0–999.0) | 891.5 (648.0–1023.4) |

Suppl. Table 2: longitudinal data of F64L, V30M and all non-F64L cohorts. Data are presented as median (interquartile range). Abbreviations: mBMI, modified Body Mass Index; NIS, Neuropathy Impairment Score; KPS, Karnofsky Performance Status; QoL-DN, Norfolk Quality of Life–Diabetic Neuropathy; CADT, Compound Autonomic Dysfunction Test; 6MWT, Six-Minute Walk Test; IVS, interventricular septal thickness; NYHA, New York Heart Association functional class; m, meters, mm, millimetres
